# Pregnancy in Women With Cerebral Palsy

**DOI:** 10.7759/cureus.36502

**Published:** 2023-03-22

**Authors:** H. S. Deeksha, Sandhya Pajai, Neema Acharya, Shazia Mohammad

**Affiliations:** 1 Department of Obstetrics and Gynecology, Jawaharlal Nehru Medical College, Datta Meghe Institute of Medical Science (Deemed to be University), Wardha, IND

**Keywords:** caeserian section, starvation ketoacidosis, muscle spasticity, pregnancy, cerebral palsy

## Abstract

Cerebral palsy is a permanent, non-progressive, irreversible, non-curable condition with high co-morbidities and lifelong complications. Brain lesions may be present at birth or shortly after that. It may be congenital or acquired, prenatal, or abnormal brain development. The damage to the brain is non-progressive. It mainly affects movement, coordination, strength, and posture. Cerebral palsy is believed to increase women's chance of unfavorable pregnancy outcomes. According to studies, the main outcome of cerebral palsy in pregnant women is premature birth. Secondary outcomes like LSCS, labor induction, low 5-minute APGAR, small for gestational age (SGA), large for gestational age (LGA), and stillbirth point to the necessity for increased surveillance during prenatal treatment. A 27-year-old primigravida with a known case of dystonic Cerebral palsy since childhood presented with a history of nine months of amenorrhea, pain in the abdomen, and backache for one day. Per abdominal examination, the uterus was 34 weeks in size with Breech presentation, mild contractions were present, and a fetal heart rate of 146 beats per minute, which was regular. On per-vaginal examination cervical os was one finger loose, the show was present. The patient underwent a planned Lower segment caesarean section after neuro physician and anesthesiologist clearance and delivered a healthy female baby of 2.4 kg. Both mother and baby were stable.

## Introduction

Cerebral palsy is a motor function disorder caused by insult to the developing brain, which may be due to birth asphyxia, trauma, infection, or prematurity in antenatal, perinatal, or postnatal periods. In developing nations like India, the incidence of cerebral palsy is approximately 3/1,000 live births. This has not altered recently despite better antenatal care and public health [1]. This may be due to an increase in the survival rate of premature infants due to advanced medical technologies. Cerebral palsy clinically manifests as spastic (70%), dyskinetic (10%), ataxic (10%), and mixed (10%) types according to the involvement of the brain. Additional developmental disabilities such as mental retardation, epilepsy, visual, hearing, speech, cognitive, and behavioral abnormalities, and chronic systemic diseases may be present in these patients [2]. The most common symptom is spasticity which is an involuntary muscle contraction that causes more strain on joints and throughout pregnancy gradual weight gain adds more stress, and hence mobility is limited.

These involuntary spasms worsen the process of childbirth. These spasms can increase so much that normal delivery becomes impossible, and a caesarean section is preferred. Cerebral palsy does not affect fertility and hence does not pass genetically. A mother or a father with cerebral palsy can have a normal child.

Nevertheless, associated motor dysfunction makes pregnancy more challenging. The baby may have low APGAR at 5 minutes after delivery than a baby born to a mother without cerebral palsy. Women with cerebral palsy can improve their prenatal and postnatal experiences by having access to treatments like physical and occupational therapy. Hence, with the appropriate prenatal care and attention during labor and delivery, many women with cerebral palsy can have a safe pregnancy.

## Case presentation

A 27-year-old primigravida with 35.3 weeks of gestational age with a known case of dystonic cerebral palsy presented with nine months of amenorrhea with pain in the abdomen and backache with 7-10episodes of vomiting and not tolerant to feeds for one day. Her last menstrual period was 12.5.21 with an expected date of delivery on February 16, 2022. Her period of gestation was 35.5 weeks according to her first scan. The patient attained menarche at the age of 15 years. Her past menstrual cycles were regular, with 28-30 days cycle with 3-4 days flow. She used 3-4 pads per day. There was no history of the passage of clots or dysmenorrhea during the cyclical flow.

**Figure 1:**
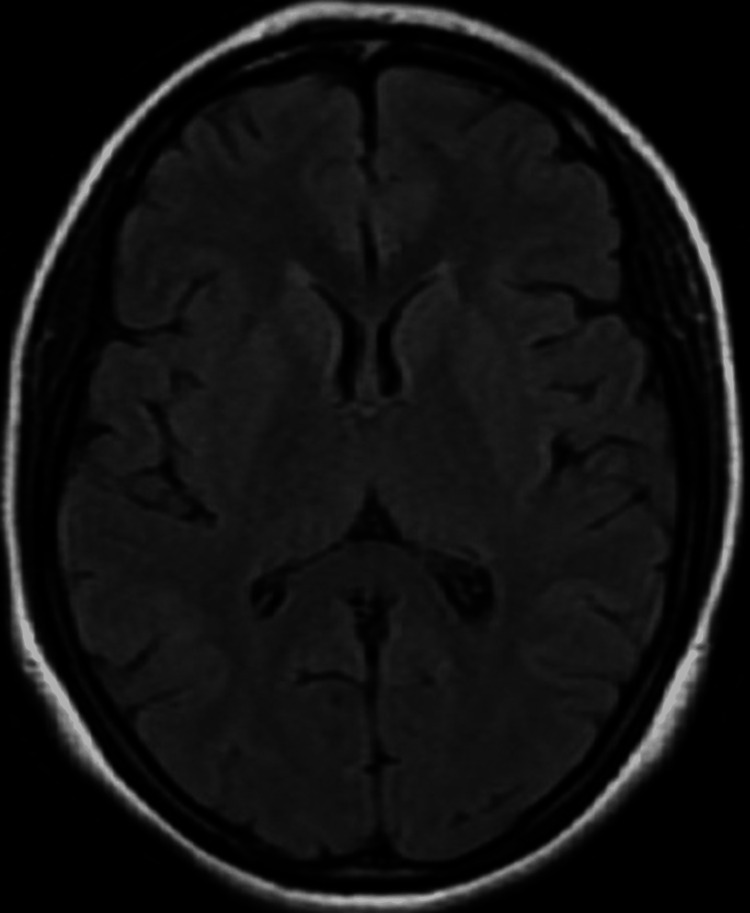
MRI of brain in T2 FLAIR: normal

**Figure 2:**
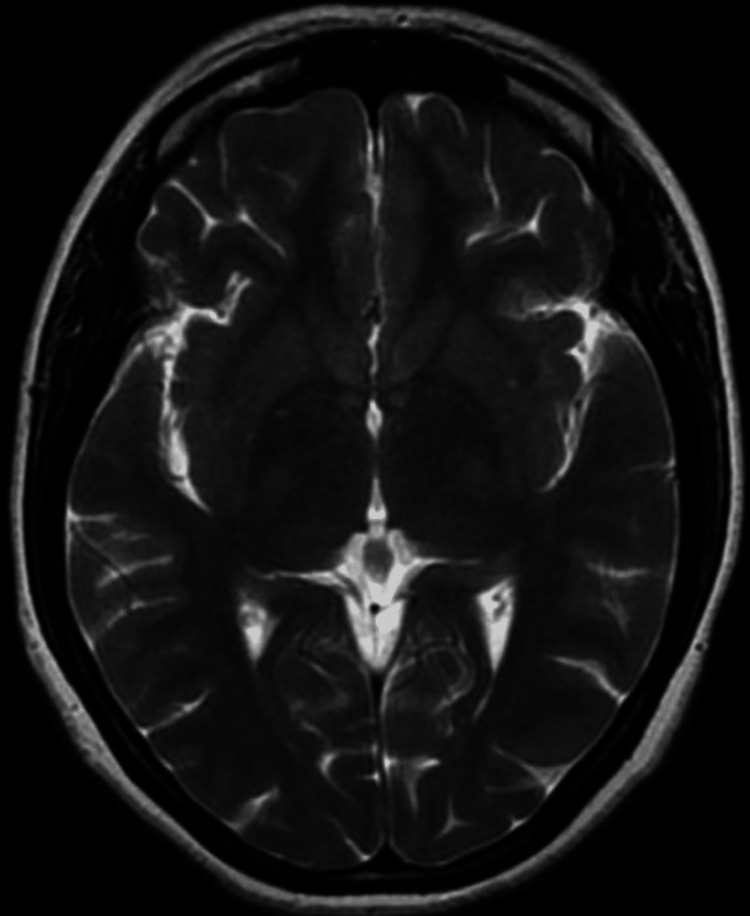
Axial T2WI shows no obvious brain parenchymal abnormality

The patient was born to parents of first-degree consanguineous marriage at 35 weeks of gestation by vaginal delivery and weighed 2 kg at birth. The baby had a delayed cry in view of three loops of cord around the neck. The post-delivery baby was resuscitated and cried after 5-6 minutes after birth. During the development of the child, her milestones were delayed, but eventually, all milestones were attained. She had good verbal communication with her family. She completed her schooling till nine grade, and she was able to perform her daily activities independently but at a slower rate. The patient belongs to an upper- lower class family with a score of 8 according to Kuppuswamy's socio-economic status scale 2022. During her childhood, she was not given any attention, regular check-ups were not done, and she was not on any medication. The patient has a history of difficulty performing mathematical calculations and has a poor memory. She had difficulty completing the tasks with poor coordination in complex and skilled work. At the age of 16 years, she got her MRI brain done in view of titubation and was reported to be normal, with no focal neurological deficits (Figures 1, 2). The age of marriage was 25 years, consanguineous, and conceived naturally after one year of marriage. There is no history of diabetes mellitus, hypertension, tuberculosis, epilepsy, or thyroid disorders in the past. There is no history of previous blood transfusions, Orofer injections, or any medication intake. The Patient also does not have any history of difficulty in walking, frequent falls, rigidity, refractory errors, or squint since childhood. There is no similar history in her Family. The Patient had normal bowel bladder habits and adequate sleep.

The pre-pregnant weight of the patient was 45 kg, and her height is 1.52 meters with a BMI of 20 kilograms per meter square. The weight of the patient at 35 weeks of gestational age is 51 kg indicating that the weight gain is less than adequate. On examination of the patient, she was alert, cooperative, and aware of time, place, and others.

On general physical examination, the patient was afebrile on touch with the temperature being 37 degrees Celsius. Her pulse rate on the left radial artery was 82 beats per minute and was regular in rate, rhythm, and volume. Blood pressure was measured in the left lateral position on the left arm and was 124/80 MMHG with a respiratory rate of 20 cycles per minute which was regular. On examination of the lower palpebral conjunctiva, there was no pallor, and the sclera was clear suggesting no icterus. There was no cyanosis, clubbing, lymphadenopathy or edema. On cardiovascular examination, S1 and S2 were normal with no murmurs and apex beat felt at the fourth intercostals space 10 cm lateral to the midline. On auscultation, bilateral air entry was equal and normal vesicular breath sounds were present with no adventitious sounds. The findings of the central nervous system are shown in Table 1.

**Table 1:** Central nervous system examination

MENTAL STATUS EXAMINATION	
Consciousness	Conscious
Orientation	Oriented to time, place and person
Speech	Normal
Language	Normal in fluency, comprehension, repetition
Memory	Immediate, recent and remote memory intact
Reasoning/judgment	Normal
CRANIAL NERVES: ALL cranial nerves	Intact
MOTOR SYSTEM	
Bulk of the muscles in all 4 limbs	Normal
Tone of the muscles: Upper limbs	Spasticity present
Lower limbs	Normal tone
Power of muscles: Upper limbs	4/5
Lower limbs	4/5
Gait	Normal
REFLEXES	GRADE
1 BICEPS [C5,C6,musculataneous nerve]	2
2 TRICEPS [C6,C7,radial]	2
3 PATELLAR [L2-L4,femoral]	2
4 ANKLE [S1 –S2,tibial]	2
SUPERFICIAL REFLXES: PLANTAR [BABINSKI]	Flexion
SENSORY EXAMINATION	
1. Pain and temperature	Normal
2. touch and pressure	Normal
3. vibration	Normal
4. proprioception: stereo gnosis	Normal
CORDINATION	
Finger nose finger test	Normal
Rapid alternative movements	Normal
Heel knee tests	Normal

On inspection of per abdomen, the line nigra was seen with stria gravidarum. On palpation uterus size was 34-36 weeks size, fundal grip was suggestive of hard ballotable mass suggesting the head, and on lateral grip there was continuous board-like rigidity on the left side suggesting the back. There were 2-3 mild contractions lasting for 20-25 seconds examined over 10 minutes. Fetal heart rate was 150 beats per minute, regular. Non-stress test was done, there was beat-to-beat variability present with more than two accelerations in 10 minutes and no decelerations. With informed consent, the patient was made comfortable on the table and per vaginal examination was done which suggested cervical OS to be 1-2 cm dilated with station of head high up. There was no show or bulging of membranes, pelvis was inadequate with prominent left ischial spine indicating the increased chances of obstructed labor. Laboratory findings are shown in Table 2.

**Table 2:** Laboratory findings

HEMOGLOBIN	11.7GM%	URINE ALB	+1
WHITE BLOOD CELLS	7800/MM3	URINE SUGAR	+1
PLATELETS	1.87L/MM3	URINE KETONES	+2
HEMATOCRIT	33.6	PUS CELLS	3-4CELLS/HPF
FASTING BLOOD SUGAR	87MG/DL	THYROID STIMULATING HARMONE	1.59
HBA1C	4.9	URIC ACID	5.2
POST PRANDIAL BLOOD SUGAR	149	SODIUM	135
RANDOM BLOOD SUGAR	94	POTASSIUM	4.4

Ultrasonography was done on February 14, 2022 and it showed single intrauterine life fetus with average gestational age of 35.4 weeks and corresponding weight of 2,495 g with liquor of 7 and placenta located anterior, grade II predominantly on maternal side with breech presentation. Doppler suggested normal umbilical artery, uterine artery and middle cerebral artery flow.

Physician opinion was taken in view of urine ketone and sugar and was diagnosed with starvation ketoacidosis and started on Injection Thiamine 100 mg IV TDS and IV fluids. Urine ketone was negative after two days of adequate hydration. After the neuro physician and anesthesiologist, clearance patient was taken up for elective lower segment caesarean section. Informed, written and valid consent was taken before the procedure and intra/postoperative risks were explained. Patient underwent elective LSCS under General Anesthesia and delivered a female baby of 2.4 kg. The baby cried immediately after birth with APGAR score of 8/10 and 9/10. The patient withstood the procedure well with no anesthetic complications. Postoperatively, the patient was observed for 24 hours, and the baby shifted to mother side. Breastfeeding was initiated within two hours. Both mother and baby were healthy.

## Discussion

Cerebral palsy is the most common motor disability in children. Research on pregnancy and neonatal outcomes in this population is scant. Any circumstance that will have an impact on the developing fetal and neonatal brain can result in cerebral palsy. Congenital abnormalities, fetal growth restriction, multiple gestations, infection during the prenatal and postnatal period, birth asphyxia, preterm delivery, untreated maternal hypothyroidism, perinatal stroke, and thrombophilia are among the significant and widespread risk factors.

Numerous clinical disorders reveal persistent posture and mobility issues. The affected person's range of motion, posture, and muscular tone are all abnormal, which limits their capacity to do anything. The severity of these challenges varies from cerebral palsy patient to cerebral palsy patient and may fluctuate over the course of a lifetime [1]. Seizures, abnormalities in sensation, perception, cognition, communication, and behavior, in addition to secondary musculoskeletal problems, are commonly present together with the motor impairments of cerebral palsy.

Despite having positive clinical symptoms, patients with cerebral palsy may have normal brain scan results. According to studies, one in nine biological children and roughly 30% of people with cerebral palsy live with a partner [2]. Although cerebral palsy is not a disease that progresses on its own, its clinical appearance may as the brain develops [3]. Due to a variety of factors, pregnant women with cerebral palsy find it challenging to receive the right antenatal care [4]. We anticipated that cerebral palsy patients who became pregnant would be more likely to encounter unfavorable circumstances, such as a higher risk of falling because of their impaired balance and coordination, which are made worse by changes in the center of gravity as the pregnancy advances [4]. Physical therapists can support women in maintaining or regaining strength and balance and might suggest wheelchairs or walkers utilize during times of limited mobility [4]. Increased neonatal mortality and morbidity, as well as fetal maturation, are all highly correlated with preterm birth [1,2]. We choose preterm birth as the main outcome. Maternal morbidity, low BMI, stress (both physiological and psychological), smoking, preeclampsia, and gestational diabetes are all associated with preterm birth.

Pregnant women with Cerebral palsy may experience high-stress levels due to psychosocial worries and physical difficulties brought on by pain and spasticity [1,2]. The curvature of the spine frequently makes spinal anesthesia more challenging in patients with cerebral palsy. We also had concerns related to one of the case reports suggesting that spinal anesthesia removes inhibition of athetoid movements leading to increased athetoid movements that are harmful to the mother and baby in-utero. Hence general anesthesia is preferred. Generally, an epidural is not given as they worsen the spasm.

Cerebral palsy per se does not affect contractions and cervical dilatations. Sometimes abnormal, involuntary spasms make standard delivery impossible, and a lower segment caesarian section becomes necessary. National health services (NHS) advise avoiding any medications during the first three months of pregnancy. Patients' family often neglects patient with Cerebral palsy and hence are prone to malnutrition. In our case, the patient did not gain enough weight during her pregnancy and also suffered from starvation acidosis.

Optimally, women with cerebral palsy should enhance their physical and mental health before becoming pregnant [4]. Following the delivery, rehabilitation is recommended to return to pre pregnant functional state [4]. And longer follow-up is advised to determine if she has attained the pre-pregnant functional state. Given this greater risk and the added burden of parenting a child with a disability, women with Cerebral Palsy are more likely to have postpartum depression [4,5]. So, it is recommended to get good mental healthcare.

## Conclusions

We find that the patient with cerebral palsy has a higher risk of an unfavorable pregnancy outcome. The risk of hyperemesis, anemia, preterm birth, gestational diabetes, hypertensive disorders of pregnancy, and admission to high-risk pregnancy units seems to be higher in women with cerebral palsy. Showing that this group requires additional monitoring during antenatal care, preferably provided by an obstetrician who is knowledgeable about Cerebral palsy and its issues in collaboration with a neurologist. It is important to properly address and manage pregnancy complications, physical issues, discomfort, skeletomuscular issues, and psychological issues. With the right care, it is possible to achieve the goal of a healthy mother and infant.
